# School-based mental health support for students with intellectual disability in the Middle East and North Africa: a 26-year scoping review

**DOI:** 10.3389/fpsyg.2026.1896262

**Published:** 2026-10-15

**Authors:** Majed Alshehri

**Affiliations:** Department of Special Education, College of Education, Prince Sattam bin Abdulaziz University, Alkarj, Saudi Arabia

**Keywords:** intellectual disability, MENA, mental health support, school-based, scoping review, special education

## Abstract

Students with intellectual disability (ID) in the Middle East and North Africa (MENA) region face significant mental health challenges, yet the extent and nature of school-based support for them remain unclear. This scoping review mapped the English-language evidence on school-based mental health support for this population. Six sources were searched for studies published between January 2000 and May 2026. The initial search identified 255 records, of which 210 were screened, and an update search identified one further study. Six studies were included; two met all eligibility criteria and four met them in part. One study evaluated an intervention, Cognitive-Behavioral Play Therapy, and reported improved self-esteem; it was delivered in residential welfare centers rather than schools. One study reported a strong association between bullying victimization and depressive symptoms among students with mild ID. Four studies surveyed 1,037 practitioners in Saudi Arabia, Qatar, and the United Arab Emirates, three of them in mixed disability populations. Practitioners reported moderate and uneven preparedness in behavioral assessment, social skills training, trauma-informed practice, and mental health support, and self-efficacy and perceived school support were associated with teachers’ attitudes toward supporting student mental health. No school-delivered intervention was identified, and no included study reported follow-up or a family component. Overall, the English-language evidence identified by this review is small, heterogeneous, and geographically concentrated, and few students with ID took part directly. The findings point to a need for culturally adapted school-based interventions, stronger mental health content in teacher preparation, and explicit inclusion of students with ID in regional education and mental health frameworks.

**Systematic review registration:**

https://doi.org/10.17605/OSF.IO/HJQMR.

## Highlights

- One of the first reviews of school mental health support for students with ID in MENA.
- Six studies were identified in the English-language literature from 2000 to 2026.
- The only intervention study was delivered in residential centres, not schools.
- Teacher preparedness for student mental health support was limited.
- Major gaps exist in culturally adapted school-based interventions.

## 1 Introduction

Intellectual disability (ID) is among the most prevalent neurodevelopmental conditions globally, affecting approximately 1%–3% of the school-age population, with higher rates documented in low- and middle-income countries where early detection infrastructure remains limited (Maulik et al., 2011; Emerson and Llewellyn, 2022). Common comorbidities include anxiety disorders, attention-deficit/hyperactivity disorder, depressive disorders, and oppositional defiant disorders, each compounding the developmental, social, and educational difficulties already associated with ID (Einfeld et al., 2011; Buckley et al., 2020). Untreated, these comorbidities persist across the lifespan and contribute to school exclusion, family stress, and diminished long-term outcomes (Totsika et al., 2022; Patel et al., 2010; Sendmaa et al., 2024; Sevgi and Ayran, 2024). Up to half of all children and adolescents with intellectual disability meet criteria for a diagnosable psychiatric disorder, a rate three to four times that of their peers (Totsika et al., 2022; Buckley et al., 2020); however, access to school-based mental health interventions remains limited for many of these individuals.

For most children with ID, schools are the primary developmental setting and a key platform for early identification and psychosocial support, particularly where specialist services are limited or geographically inaccessible (Windsor et al., 2023). Yet the international literature on school-based mental health interventions for this population remains largely concentrated in high-income Western contexts. Windsor et al.’s (2023) systematic review identified 27 eligible randomized controlled trials drawn almost entirely from North America, Europe, and Australia. The closest review to address the Global-South gap, Alpuche De Lille et al. (2025), examined low- and middle-income countries and found only five studies from the entire Eastern Mediterranean region across 26 years, with limited representation of students with intellectual disability in school-based settings.

The Middle East and North Africa (MENA) region presents a significant gap in evidence and service provision. Mental health infrastructure across many MENA countries remains limited relative to population need (Charara et al., 2017; Rahman, 2018; Okasha et al., 2025). Child and adolescent services are often concentrated in urban tertiary settings, making them inaccessible to many families (Abdalhai et al., 2023; Okasha et al., 2025). Stigma surrounding both disability and mental illness continues to constrain help-seeking behaviors in the region (Zuberi et al., 2021; Medabesh et al., 2024).

The WHO Eastern Mediterranean Regional Office’s school mental health framework, developed across 10 MENA countries, has a limited focus on disability-specific populations (Hamoda et al., 2022; Khoo et al., 2024). The result is a notable gap in both research evidence and policy attention for students with intellectual disability in the region.

In this review, school-based mental health support refers to any promotion, prevention, identification, or intervention activity that addresses the emotional, behavioral, or social wellbeing of students with ID and is delivered within, or organised through, an educational setting, together with the workforce capacity needed to deliver it. The construct has three components, which the review maps separately: interventions evaluated with students, the mental health needs that such support would target, and the knowledge and preparedness of the practitioners expected to provide it.

This scoping review aims to map the available English-language evidence on school-based mental health support for students with ID in the MENA region and to identify gaps in that evidence. It was conducted in accordance with JBI methodology and is reported using PRISMA-ScR (Tricco et al., 2018). The MENA region is defined here as the 22 member states of the League of Arab States, together with Iran and Turkey. To our knowledge, this is one of the first reviews to examine this intersection within the MENA context. The review addresses three research questions:

1 What is the nature and extent of empirical evidence on school-based mental health support for students with intellectual disability in MENA countries?
2 What mental health outcomes, needs, and intervention effects have been documented for students with intellectual disability in MENA school settings?
3 What is the state of practitioner knowledge, preparedness, and implementation capacity for school-based mental health support for students with intellectual disability in the MENA region?

## 2 Methods

### 2.1 Study design

This study employed a scoping review design following the framework outlined in the JBI Manual for Evidence Synthesis (Peters et al., 2020). It is reported in accordance with the Preferred Reporting Items for Systematic Reviews and Meta-Analyses extension for Scoping Reviews (PRISMA-ScR; Tricco et al., 2018). A scoping review was selected as the appropriate methodology because the primary aim is to map the breadth, nature, and distribution of available evidence rather than to synthesize effect sizes or establish causal claims. This approach is suited to subjects with heterogeneous study designs, diverse interventions, and an emerging rather than established evidence base (Munn et al., 2018). No quality assessment of included studies was conducted, consistent with JBI scoping review conventions (Peters et al., 2020). The review protocol was preregistered on the Open Science Framework before database searching began (doi:10.17605/OSF.IO/HJQMR); deviations from the registered protocol are reported in Section 2.2.

### 2.2 Eligibility criteria

Eligibility criteria were defined using the Population, Concept, and Context (PCC) framework recommended for scoping reviews (Peters et al., 2020) and are presented in Table 1. Two groups were eligible as participants: students with ID, and school-based practitioners reporting on their practice with students with ID. A study was considered school-based if the support it examined was delivered in, or organised through, a day school, special school, or educational centre serving students with ID. Residential welfare and clinical settings did not meet this definition.

**Table 1:** Eligibility criteria using the PCC framework.

Component	Inclusion	Exclusion
Population	(a) Children and adolescents aged 3–18 with confirmed or reported intellectual disability or intellectual developmental disorder; or (b) school-based practitioners (e.g., teachers, paraeducators, school psychologists) reporting on their practice with students with ID.	Studies focused exclusively on caregivers or typically developing students; practitioner studies with no reference to students with ID.
Concept	School-based mental health interventions, psychosocial support, social–emotional learning, behavioral support, psychological therapies, or counselling programs; studies documenting the mental health needs of students with ID in school settings; studies examining practitioner knowledge or preparedness regarding mental health support for students with ID.	Interventions delivered exclusively in clinical or community settings; physical health or medical interventions unrelated to mental health.
Context	MENA countries, defined as the 22 member states of the League of Arab States (Algeria, Bahrain, Comoros, Djibouti, Egypt, Iraq, Jordan, Kuwait, Lebanon, Libya, Mauritania, Morocco, Oman, Palestine, Qatar, Saudi Arabia, Somalia, Sudan, Syria, Tunisia, the United Arab Emirates, and Yemen), together with Iran and Turkey.	Studies conducted outside the defined MENA geographic scope.
Study types	All empirical designs including RCTs, quasi-experimental, single-case, qualitative, mixed-methods, and descriptive surveys; peer-reviewed articles, theses, and institutional reports.	Opinion pieces, editorials, theoretical papers without empirical data, and studies for which full text could not be retrieved.
Language and date	English-language publications, January 2000 – May 2026.	Non-English publications; publications outside the specified date range.

Five changes were made to the registered protocol. First, Scopus, listed in the protocol, was unavailable for article-level searching when the searches were run, and ProQuest Dissertations & Theses Global was searched in its place to capture doctoral research. Second, the five registered research questions were consolidated into the three reported above. Third, the registered research questions covered intervention studies and studies of practitioner knowledge and preparedness. During full-text screening, it became clear that studies documenting the mental health needs of students with ID in school settings formed part of the available evidence, and the Concept criterion was expanded to include them; the review title was changed from “interventions” to “support” accordingly. Iterative refinement of this kind is accepted in scoping reviews provided it is reported transparently (Arksey and O’Malley, 2005; Levac et al., 2010; Peters et al., 2020). Fourth, the registered search period ended in March 2026 and was extended to May 2026 through an update search using the same strategy. Fifth, the protocol specified independent verification of a random 20% sample of screening decisions; this was not carried out by a second reviewer. The fit of each included study against the final criteria is rated in Table 2.

**Table 2:** Summary of studies.

Author(s), Year	Country	Design	Setting	Participants	Focus / intervention	Measures	Key findings	Theoretical framework	Eligibility fit and scope notes
Alharthi et al. (2026)	Saudi Arabia (Mecca)	Descriptive correlational–comparative	Inclusive public primary schools	*N* = 68 students with mild ID 28 males, 40 females Aged 7–14 years IQ 55–70 (school records)	No intervention. Examined association between bullying victimization and depressive symptoms; gender differences	Perceived Depression Symptoms Scale (35 items, α = 0.92) Bullying Victim Scale (23 items, α = 0.93) Student self-report with teacher facilitation	Bullying–depression: *r* = 0.708, *p* < 0.05 Females scored significantly higher on all bullying and depression dimensions (*p* < 0.05) All subscale correlations positive and significant (0.481–0.653)	Beck’s Cognitive Theory of Depression Bronfenbrenner’s Ecological Systems Theory	Full fit. Correlational design with no intervention or practitioner component; included as documentation of mental health needs under the Concept criterion.
Bana et al. (2017)	Iran (Tehran)	Quasi-experimental pre–post with control group	Welfare Organization boarding centers for children with special needs	*N* = 40 children with educable ID Intervention: *n* = 20 Control: *n* = 20 Aged 8–12 years IQ 50/55–70 (Raven’s CPM)	Cognitive-Behavioral Play Therapy (CBPT) 12 sessions × 60 min, 2×/week Group format (subgroups of 5) Researcher-delivered (CBPT-licensed)	Cooper-Smith Self-Esteem Inventory, School Short-Form (58 items, α = 0.64) Researcher-administered Raven’s CPM (screening only)	Intervention group: pre M = 12.90 → post M = 26.95 Control group: pre M = 12.10 → post M = 12.40 t = 13.434, df = 38, *p* < 0.001 Mean difference = 14.55	Cognitive-Behavioral Therapy (developmental adaptation) CBPT framework	Partial fit (setting). Residential welfare centres providing educational programming; the setting does not meet the school-based definition. Retained as related contextual evidence.
Al-Hadad (2017)	Qatar (Doha)	Descriptive analysis (survey + document analysis)	Shafallah Centre for Children with Special Needs (specialized educational facility)	Survey: N = 168 professionals 86 special educators, 65 paraeducators, 13 psychologists, 4 support staff 63 males, 105 females Document analysis: 10 FBA/BIP records	No intervention. Assessed FBA/PBS practices, practitioner skill levels, and professional development needs	Special Education In-Service Needs Assessment PBS Implementation Survey FBAs/BIPs Analysis Rating Scale Demographic Survey Arabic translated (forward–back)	FBA/BIP technical adequacy: unsatisfactory All FBAs developed by single practitioners (no team approach) 40% of BIPs failed to link function to intervention Lowest skill: hypothesis testing (M = 2.89/4) Top PD need: assistive technology (M = 2.93), FBA (M = 2.80)	Positive Behavior Support / PBIS Applied Behavior Analysis Response to Intervention	Partial fit (population). Students served include ID, ASD, and multiple disabilities; results not reported separately for ID. Single-site study. Data collected June 2011; dissertation published 2017.
Alshehri (2021)	Saudi Arabia (nationwide)	Cross-sectional descriptive survey	Public (60.3%), private (34.6%), and charity (5%) special education schools across all 13 KSA regions	N = 182 special education teachers 151 females, 31 males 79.9% bachelor’s, 20.9% graduate degree 45.1% < 5 years’ experience	No intervention. Assessed teacher knowledge and use of social skills training strategies (visual supports, Social Stories, power cards, video modelling, role play, self-video modelling) and delivery methodology (traditional vs. technology)	Researcher-developed online survey (5 sections) Section 1: α = 0.71 Section 2: α = 0.75 Section 3: α = 0.80 Teacher self-report Arabic (back-translated)	Overall knowledge: M = 3.25/5 Visual supports highest (M = 3.90); self-video modelling lowest (M = 3.21) Traditional > technology delivery Females > males in knowledge (*p* = 0.004) Education level and experience: NS	Evidence-based practices framework (NCAEP)	Partial fit (population and concept). Population includes students with ID and/or ASD, not reported separately; social skills training framed as pedagogy and included under psychosocial support.
Alshehri (2026)	Saudi Arabia (multi-region)	Cross-sectional explanatory survey with CFA and SEM mediation	Schools under the General Directorate of Special Education across multiple Saudi regions (cluster sampling)	*N* = 443 special education teachers of students with ID 329 males (74.3%), 114 females (25.7%) 29.1% bachelor’s, 44.0% master’s, 26.9% PhD 32.1% with 1–5 yrs., 44.2% with 6–10 yrs., 23.7% with 11–15 yrs. experience	No intervention. Assessed teacher awareness of student mental health, self-efficacy for supporting mental health, perceived school support, and attitudes toward supporting student mental health	Researcher-developed questionnaire informed by the School Mental Health Self-Efficacy Teacher Survey and mental health literacy tools 4 constructs, 5-point Likert Cronbach’s α = 0.79–0.83 CR = 0.82–0.88; AVE = 0.52–0.61 CFA: χ^2^/df = 2.8, CFI = 0.95, TLI = 0.94, RMSEA = 0.07	Overall means moderately positive: Awareness M = 3.36, Attitudes M = 3.57, Self-Efficacy M = 3.65, School Support M = 3.43 Strongest correlation: Self-Efficacy ↔ School Support (*r* = 0.625, *p* < 0.01) Awareness (β = 0.118, *p* = 0.005) and School Support (β = 0.541, *p* < 0.001) jointly predicted Attitudes (R^2^ = 0.36) Self-Efficacy partially mediated effects of Awareness (β indirect = 0.015) and School Support (β indirect = 0.096) on Attitudes Female > male on awareness, self-efficacy, school support; PhD > Master’s/Bachelor’s; 11–15 yrs. experience > less experienced groups	Bandura’s Social Cognitive Theory Mental Health Literacy framework	Full fit. ID-specific population (teachers of students with intellectual disabilities); national multi-region cluster sample. Authored by the author of this review.
Opoku et al. (2024)	UAE (Abu Dhabi emirate)	Cross-sectional survey	22 public (*n* = 8) and private (*n* = 14) schools in Abu Dhabi	*N* = 244 regular classroom teachers 63 males, 181 females 89 Emirati, 154 expatriate 76% master’s degree 86% no trauma training	No intervention. Assessed perceived competence in trauma-informed practices (TIP) for students with disabilities using SAMHSA model	Teacher Trauma Management Scale (TTMS; 19 items, 6 subscales, α = 0.89) BIDR-16 social desirability scale (α = 0.60) Teacher self-report (Arabic/English) Online survey via Google Forms	Overall TIP: M = 3.60/5 (neutral/ambivalent) Trustworthiness lowest (M = 3.30); safety highest (M = 3.76) Training strongest predictor (β = −0.56, *p* = 0.001) Full model R^2^ = 0.51 Younger > older teachers (*p* = 0.001) Public > private schools (*p* = 0.004)	SAMHSA Trauma-Informed Care Framework (6 domains)	Partial fit (population). Addresses all disability types; the population served includes students with ID, but findings are not disaggregated by diagnosis.

ID, intellectual disability; ASD, autism spectrum disorder; CBPT, Cognitive-Behavioral Play Therapy; CPM, Colored Progressive Matrices; FBA, functional behavior assessment; BIP, behavior intervention plan; PBS, positive behavior support; PBIS, positive behavioral interventions and supports; PD, professional development; TIP, trauma-informed practices; SAMHSA, Substance Abuse and Mental Health Services Administration; TTMS, Teacher Trauma Management Scale; BIDR, Balanced Inventory of Desirable Responding; NCAEP, National Clearinghouse on Autism Evidence and Practice; CFA, confirmatory factor analysis; SEM, structural equation modelling; CR, composite reliability; AVE, average variance extracted; NS, not significant; M, mean; α, Cronbach’s alpha. Full fit = meets all eligibility criteria in Table 1; partial fit = does not meet one or more criteria (reason in parentheses) and is retained as related evidence. Instrument and framework sources are as cited in the original studies.

### 2.3 Information sources and search strategy

The initial searches were run in April 2026 in six sources: PubMed/MEDLINE; ERIC and APA PsycINFO, both accessed through the EBSCOhost platform; Web of Science Core Collection; ProQuest Dissertations & Theses Global; and Google Scholar. PubMed/MEDLINE covered biomedical and disability research, ERIC covered education and special education, and APA PsycINFO covered psychology and behavioral intervention. Web of Science provided multidisciplinary coverage, and ProQuest Dissertations & Theses Global and Google Scholar were used to capture doctoral research and material not indexed elsewhere. An update search using the same strategy was run in May 2026. All searches were limited to English-language publications from January 2000 to May 2026. Search strings combined four conceptual blocks with Boolean operators: (1) intellectual disability terminology, (2) mental health intervention terminology, (3) school and educational setting terminology, and (4) MENA country and regional terminology. The full search strategy is presented in Table 3. All 146 records retrieved from Google Scholar were exported and screened in full, which was within the preregistered stopping rule of screening the first 200 results per search string.

**Table 3:** Full search strategies employed for online databases.

Component	Details
Database	PubMed/MEDLINE; ERIC and APA PsycINFO (EBSCOhost); Web of Science Core Collection; ProQuest Dissertations & Theses Global; Google Scholar
Block 1: Intellectual disability	“intellectual disability*” OR “intellectual developmental disorder” OR “cognitive disability*” OR “mental retardation” OR “developmental disability*”
Block 2: Mental health intervention	“mental health” OR “psychosocial intervention*” OR “behavioral intervention” OR “anxiety” OR “depression” OR “social skills training” OR “psychological therapy” OR “psychotherapy” OR “counseling” OR “counselling” OR “social–emotional learning” OR “emotional regulation” OR “mental disorder” OR “wellbeing” OR “behavior* support”
Block 3: School setting	“school” OR “special education” OR “classroom” OR “school-based” OR “inclusive education” OR “special school” OR “educational setting” OR “resource room”
Block 4: MENA geography	“Middle East” OR “North Africa” OR “MENA” OR “Arab” OR “Saudi Arabia” OR “Egypt” OR “Jordan” OR “Lebanon” OR “Morocco” OR “Tunisia” OR “Iraq” OR “Palestine” OR “Kuwait” OR “UAE” OR “Qatar” OR “Bahrain” OR “Oman” OR “Yemen” OR “Libya” OR “Algeria” OR “Sudan” OR “Syria” OR “Iran” OR “Turkey” OR “Eastern Mediterranean” OR “Gulf”
Exclusions (applied to all databases)	NOT: mothers OR fathers OR caregivers OR memantine OR “oral health” OR “Global Burden of Disease”

Blocks 1–4 were combined using AND. Within each block, terms were combined using OR. Field tags were adapted to each database’s indexing syntax: [Title/Abstract] for PubMed; TI/AB for ERIC and PsycINFO; TS = for Web of Science; ProQuest and Google Scholar searches used equivalent free-text terms without field-specific tags. Date limits (January 2000 – May 2026) and English-language filters were applied in all databases.

The search also excluded records containing the terms mothers, fathers, caregivers, memantine, oral health, and Global Burden of Disease. These terms were intended to reduce the number of records on caregiver burden, pharmacological treatment, dental health, and population-level disease burden, none of which met the eligibility criteria. The caregiver terms may, however, also have removed school-based studies that included a family component; this is considered in Sections 4 and 4.1. In addition, the geographic block did not include Mauritania, Somalia, Djibouti, or Comoros, or the variant terms Emirates, Saudi, and Türkiye, so studies indexed only under those names may have been missed.

### 2.4 Study selection and data charting

Records from all databases were exported, and duplicates were removed by matching normalised titles before screening. Title and abstract screening and initial full-text screening were assisted by large language model tools, including Claude (Anthropic), which applied the prespecified eligibility criteria to each record. Final eligibility decisions were made by the author. All records classified as uncertain at the title and abstract stage were retained for full-text review. Full texts were retrieved for all records that passed title and abstract screening, and each was assessed against the eligibility criteria, with reasons for exclusion recorded. No second reviewer independently verified screening or charting decisions (see Section 4.1). One included study (Alshehri, 2026) was authored by the author of this review; it was screened and charted using the same criteria and charting form as all other studies. The selection process is reported using the PRISMA-ScR flow diagram (Tricco et al., 2018).

Data were extracted from all included studies using a standardized charting form. Charting was assisted by the same language model tools, and the charted data were reviewed by the author against the full texts. The following variables were captured: author(s) and year; country; study design; participant characteristics (including age range, gender, ID severity, and sample size); intervention name and type; intervention components and theoretical basis (where available); delivery format and setting; session duration and frequency; primary implementer(s) and professional role; outcome domains assessed; outcome measures and instruments used; and key findings relevant to each research question. Data were synthesized narratively using directed content analysis guided by the three prespecified research questions. Frequencies of study characteristics were reported descriptively. No meta-analysis was conducted because the evidence base was heterogeneous and exploratory, consistent with JBI scoping review methodology (Peters et al., 2020).

## 3 Results

### 3.1 Search results and study selection

The study selection process is summarised in the PRISMA-ScR flow diagram (Figure 1; Tricco et al., 2018). The initial search identified 255 records: PubMed/MEDLINE (*n* = 15), ERIC (*n* = 33), ProQuest Dissertations & Theses Global (*n* = 5), Web of Science (*n* = 34), APA PsycINFO (*n* = 22), and Google Scholar (*n* = 146). After 45 duplicates were removed, 210 titles and abstracts were screened and 193 were excluded. All 17 remaining reports were retrieved and assessed in full. Twelve were excluded because the study was not conducted in a MENA country (*n* = 3), the full text was available only in Persian despite an English-language abstract (*n* = 3), the intervention was delivered to caregivers only (*n* = 2), the setting was not school-based (*n* = 2), the report was a study protocol without data (*n* = 1), or the report duplicated an included study (*n* = 1). Five studies were retained from the initial search. The update search in May 2026 retrieved these five studies again and identified one additional eligible study (Alshehri, 2026), giving six included studies.

**Figure 1:**
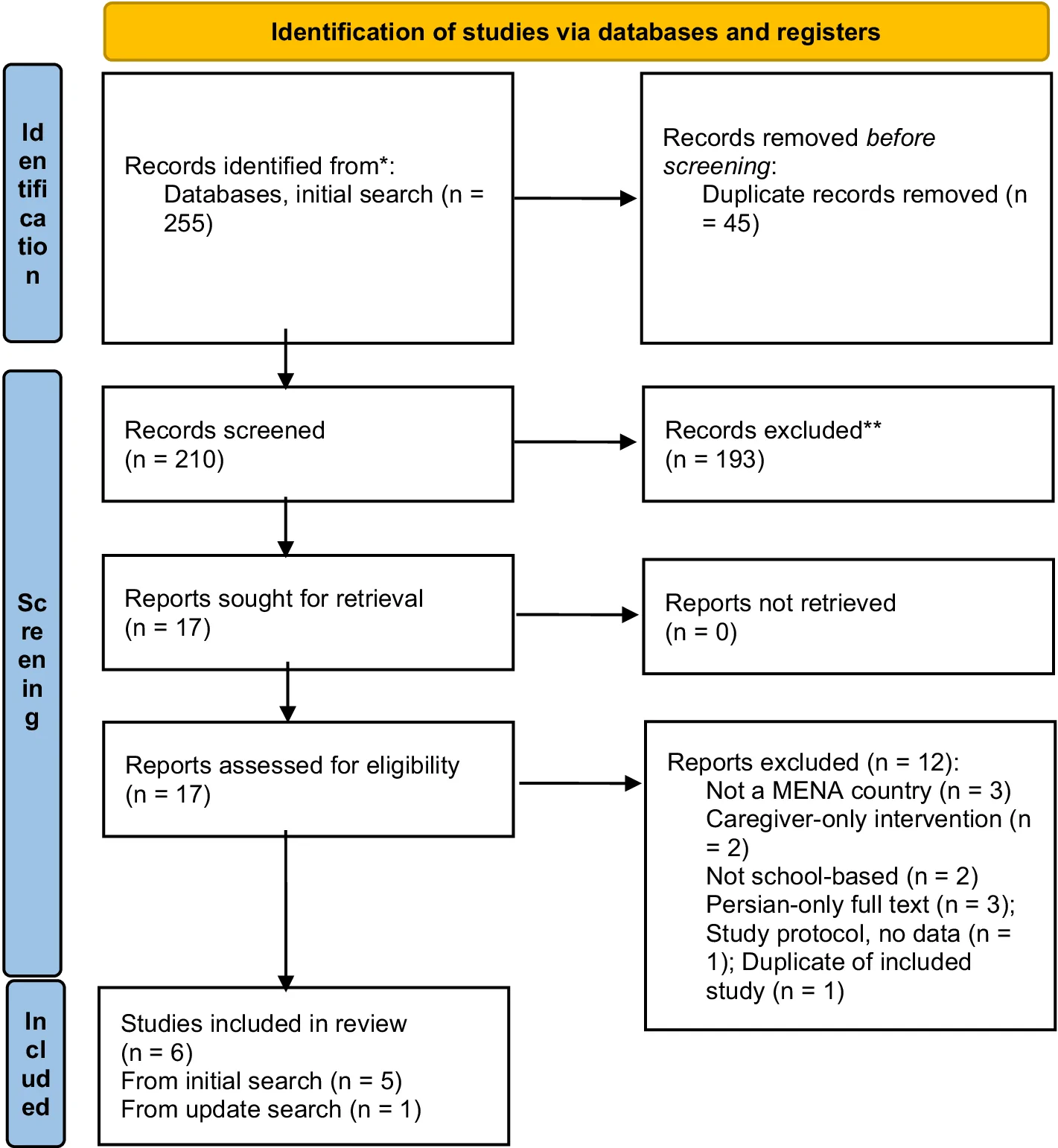
PRISMA-ScR flow diagram of the study selection process. The initial search identified 255 records; after removal of 45 duplicates, 210 titles and abstracts were screened and 17 reports were assessed in full, of which 5 were included. An update search in May 2026 identified one additional eligible study, giving six included studies. Reasons for full-text exclusion are shown.

Two studies met all eligibility criteria (Alharthi et al., 2026; Alshehri, 2026). Four met them in part: one intervention study was delivered in residential welfare centres rather than schools (Bana et al., 2017), and three practitioner studies drew on mixed disability populations in which results for students with ID were not reported separately (Al-Hadad, 2017; Alshehri, 2021; Opoku et al., 2024). These four studies were retained as related evidence. The fit of each study is rated in Table 2, and findings that rest only on partial-fit studies are identified as such in the text.

### 3.2 Nature and extent of empirical evidence

The six included studies were published between 2017 and 2026 and were conducted across four MENA countries: Saudi Arabia (*n* = 3), Iran (*n* = 1), Qatar (*n* = 1), and the United Arab Emirates (*n* = 1). Four studies were published as peer-reviewed journal articles (Alharthi et al., 2026; Alshehri, 2026; Bana et al., 2017; Opoku et al., 2024) and two were doctoral dissertations (Al-Hadad, 2017; Alshehri, 2021). The characteristics of each study, including settings, design, participants, focus, measures, and key findings, are summarized in Table 2.

Collectively, the six studies encompassed 1,145 participants, though the composition of these samples was heterogeneous. Only 108 individuals were students with intellectual disability who served as direct participants, 68 in Alharthi et al. (2026) and 40 in Bana et al. (2017), while the remaining 1,037 were school-based practitioners. Alharthi et al. (2026) assessed 68 students with mild ID (aged 7–14, 28 males and 40 females) enrolled in inclusive primary schools in Mecca, Saudi Arabia, and Bana et al. (2017) delivered a play therapy intervention to 40 children with educable ID (aged 8–12, IQ 50–70) in Welfare Organization boarding centers in Tehran, Iran. In contrast, the remaining four studies recruited only school-based professionals. Al-Hadad (2017) surveyed 168 special education professionals and analyzed 10 functional behavior assessment documents at the Shafallah Centre for Children with Special Needs in Doha, Qatar; Alshehri (2021) surveyed 182 special education teachers across all 13 administrative regions of Saudi Arabia; Alshehri (2026) surveyed 443 special education teachers across multiple Saudi regions through cluster sampling under the General Directorate of Special Education; and Opoku et al. (2024) surveyed 244 regular classroom teachers from 22 schools in the Emirate of Abu Dhabi, UAE.

The methodological scope of the included studies was narrow. One study employed a quasi-experimental pre–post design with a control group (Bana et al., 2017), one used a descriptive correlational–comparative design (Alharthi et al., 2026), three used cross-sectional descriptive surveys (Al-Hadad, 2017; Alshehri, 2021; Opoku et al., 2024), and one used a cross-sectional explanatory survey incorporating confirmatory factor analysis and structural equation modelling (Alshehri, 2026). Only one study additionally incorporated document analysis as a supplementary data source (Al-Hadad, 2017). No randomized controlled trial, single-case experimental design, longitudinal design, qualitative study, or mixed-methods study was identified in the final corpus.

Sample sizes ranged from 40 (Bana et al., 2017) to 443 (Alshehri, 2026). All studies used convenience, purposive, or cluster sampling, and four of six were conducted at a single site or drew from a single institutional context. Two studies achieved broader geographic coverage in Saudi Arabia (Alshehri, 2021; Alshehri, 2026). However, Alshehri (2021) reported a low completion rate (31.8%) and used social media-based recruitment, introducing self-selection concerns. Similarly, Alshehri (2026) relied partly on school principals to circulate the survey link, which may have introduced selection bias, as acknowledged in the study.

Of the six included studies, only one (16.7%) implemented and evaluated a specific mental health intervention for students with intellectual disability: Bana et al. (2017) delivered a Cognitive-Behavioral Play Therapy (CBPT) programme to children with educable ID in residential welfare centres in Iran. Because this setting does not meet the review’s definition of school-based, the study is treated as related contextual evidence rather than as a school-based intervention study. The CBPT programme comprised 12 sixty-minute group sessions delivered twice weekly over 6 weeks. The intervention was grounded in cognitive-behavioral therapy adapted for developmental appropriateness, using play as a medium to communicate and indirectly teach empirically supported techniques. Session content included structured play activities targeting self-awareness, problem-solving, cooperation, emotion expression, fine motor skills, and self-esteem enhancement through games such as puzzles, Lego construction, bowling, playdough, and creative arts. The programme was delivered to small groups of five children within the intervention group of 20.

The remaining five studies did not implement interventions but documented the conditions under which interventions would need to operate. Alharthi et al. (2026) demonstrated a strong positive correlation (*r* = 0.708, *p* < 0.05) between bullying victimization and depressive symptoms among students with mild ID, with female students exhibiting significantly higher levels across all measured domains. The four practitioner-knowledge studies (Al-Hadad, 2017; Alshehri, 2021; Alshehri, 2026; Opoku et al., 2024) examined the infrastructure of professional preparedness rather than specific intervention programmes. They documented what school-based personnel know, can do, and perceive as areas of professional development need in relation to behavioral support, social skills training, trauma-informed practices, and direct mental health support for students with disabilities.

Within the English-language literature identified by this search (2000–2026), no school-delivered mental health intervention for students with ID was found in the MENA region. The only intervention study identified, a quasi-experimental evaluation of a programme targeting self-esteem, was delivered in residential welfare centres. No study evaluated school-based interventions addressing anxiety, depression, social–emotional learning, counselling, or behavioral support specifically designed for students with ID in MENA school settings.

### 3.3 Mental health outcomes, needs, and intervention effects

Outcome and needs data were available from two studies that directly assessed student-level variables. Bana et al. (2017) measured self-esteem using the Cooper-Smith Self-Esteem Inventory (School Short-Form, 58 items) and reported that the CBPT intervention group’s self-esteem scores more than doubled from pre-test (M = 12.90, SD = 2.97) to post-test (M = 26.95, SD = 3.63), while the control group showed negligible change (pre-test M = 12.10; post-test M = 12.40). The between-group difference was statistically significant (*t* = 13.434, df = 38, *p* < 0.001, mean difference = 14.55). No standardized effect size was reported by the authors, and the outcome instrument’s internal consistency in the study was below conventional thresholds (α = 0.64), with no follow-up assessment to evaluate the durability of the effect.

Alharthi et al. (2026) measured depressive symptoms across four dimensions (emotional, cognitive, physical, behavioral) using the Perceived Depression Symptoms Scale for Students with Intellectual Disabilities (35 items, α = 0.92) and bullying victimization across three dimensions (physical, verbal, social) using the Bullying Victim Scale (23 items, α = 0.93). Both instruments were administered as student self-reports facilitated by trained special education teachers. The overall correlation between total bullying and total depression was strong (*r* = 0.708, *p* < 0.05), with all subscale correlations positive and statistically significant, ranging from 0.481 (physical bullying and behavioral symptoms) to 0.653 (total bullying and emotional symptoms). Female students scored significantly higher than males on every bullying dimension and every depression dimension (all *p* < 0.05).

Diagnostic confirmation of intellectual disability was imprecise across both student-level studies. Alharthi et al. (2026) relied on school records documenting IQ 55–70 without specifying the diagnostic instrument. Bana et al. (2017) re-administered Raven’s Colored Progressive Matrices to confirm IQ 50–70, though the reported group mean IQs (71.10 and 71.45) slightly exceeded the stated inclusion ceiling. Neither study used standardized diagnostic criteria such as the DSM-5 or ICD-11 to confirm intellectual disability, and neither reported adaptive behavior assessment alongside cognitive measures.

Coverage across outcome domains was uneven. No study measured outcomes across the cognitive domain in relation to mental health intervention. The emotional domain was represented by self-esteem (Bana et al., 2017) and depressive symptoms (Alharthi et al., 2026). The social domain was addressed indirectly through the practitioner surveys on social skills training knowledge (Alshehri, 2021) but not through direct student-level social outcome measurement. The behavioral domain was addressed at the system level through the technical adequacy analysis of functional behavior assessments and behavior intervention plans (Al-Hadad, 2017) but not through student-level behavioral outcome data. Of the two documented student-level needs, depressive symptoms linked to bullying victimization and low self-esteem, only the latter was the target of an evaluated intervention, and that intervention was not school-based (Bana et al., 2017). Anxiety, depression, and trauma-related needs received no intervention evidence in the corpus.

### 3.4 Practitioner knowledge, preparedness, and implementation capacity

The single intervention study was delivered by the researcher, a graduate student holding a Cognitive-Behavioral Play Therapy license, under thesis supervision at the University of Social Welfare and Rehabilitation Sciences in Tehran (Bana et al., 2017). No teacher, school counsellor, or school-based practitioner delivered the intervention. Across all six studies, no intervention was delivered by a teacher, school counsellor, or school psychologist as primary implementer.

The four practitioner-knowledge studies provided a detailed characterization of the school-based workforce in three MENA countries (Qatar, Saudi Arabia, and the UAE), including two independent Saudi samples. Only one of these studies was specific to ID (Alshehri, 2026). Al-Hadad (2017) examined practitioners serving students with ID, ASD, and multiple disabilities; Alshehri (2021) examined teachers of students with ID and/or ASD; and Opoku et al. (2024) examined teachers of students across all disability categories. Findings from these three studies are therefore not specific to students with ID. Al-Hadad (2017) reported that at the Shafallah Centre in Qatar, functional behavior assessments and behavior intervention plans were developed exclusively by psychologists (*n* = 13) working as sole practitioners, with no team-based approach. Paraeducators, who comprised 39% of the workforce (*n* = 65), held predominantly high-school diplomas, and only one of 65 had a degree in special education. In Saudi Arabia, Alshehri (2021) found that 79.9% of special education teachers held a bachelor’s degree in special education and 20.9% held a graduate degree; 45.1% had fewer than 5 years of teaching experience. In a separate national multi-region cluster sample of 443 special education teachers of students with intellectual disabilities, Alshehri (2026) reported higher academic qualification, with 44.0% holding a master’s degree and 26.9% holding a PhD; 32.1% had 1–5 years of experience, 44.2% had 6–10 years, and 23.7% had 11–15 years. In the UAE, Opoku et al. (2024) reported a highly credentialed but largely untrained workforce: 76% held master’s degrees, yet 86% had received no training in trauma-informed practices. The study also found that classroom teachers scored higher on trauma-informed competence than allied professionals such as psychologists and social workers, a pattern the authors attributed to limited access to specialized professional training.

The implementer gap is compounded by the workforce composition findings: practitioners with the most direct student contact (paraeducators, classroom teachers) reported the lowest levels of relevant knowledge and skills, while those with the highest formal qualifications (psychologists) were either not involved in team-based practice (Al-Hadad, 2017) or scored lower than classroom teachers on relevant competencies (Opoku et al., 2024).

Four studies (66.7%) directly assessed practitioner knowledge and preparedness, collectively surveying 1,037 school-based professionals across the three countries. Across these studies, practitioners reported moderate and uneven levels of knowledge, confidence, and preparedness to support the mental health needs of students with disabilities, and training in specific evidence-based approaches was often absent or limited.

In Qatar, Al-Hadad (2017) found that hypothesis testing of the purpose of problem behavior was rated the lowest skill (M = 2.89 on a 4-point scale) among 168 practitioners, with recording procedures and ongoing assessment rated similarly low (both M = 2.98). Paraeducators rated their skills as low across all eight functional behavior assessment areas, with the lowest scores for interviewing caregivers and recording procedures (both M = 2.63). The three most difficult PBS implementation practices reported were formulating hypotheses from FBA data (M = 5.27 on a 7-point scale), data interpretation (M = 4.96), and using graphs to present data (M = 4.87). The top four professional development needs identified were assistive technology (M = 2.93), functional behavior assessment (M = 2.80), effective teaching procedures (M = 2.79), and restraint procedures (M = 2.79). Document analysis of 10 FBA/BIP records revealed that all were developed by a single practitioner rather than a team, 40% failed to link the identified function to the intervention plan, and none included plans for maintenance or generalization of behavior change.

In Saudi Arabia, Alshehri (2021) reported that 182 special education teachers rated their knowledge of social skills training strategies between medium and somewhat extensive (overall M = 3.25 on a 5-point scale), with visual supports rated highest (M = 3.90) and self-video modelling rated lowest (M = 3.21). Teachers reported using traditional paper-based methods more frequently than technology-based methods. Female teachers were significantly more knowledgeable than males (M = 3.70 vs. 3.30, t(180) = 2.952, *p* = 0.004), while neither educational level (*p* = 0.239) nor years of experience (*p* = 0.342) significantly predicted knowledge. Teachers attributed their knowledge approximately equally to their preparation programmes (M = 3.1) and employer training (M = 3.39), with both sources rated only modestly above neutral.

In a separate national Saudi sample, Alshehri (2026) assessed mental-health-specific preparedness among 443 special education teachers working with students with intellectual disabilities. Teachers reported moderately positive but uneven scores across all four constructs assessed: awareness of student mental health (M = 3.36/5), attitudes toward supporting student mental health (M = 3.57/5), self-efficacy for supporting mental health (M = 3.65/5), and perceived school support for mental health (M = 3.43/5). Psychometric analysis supported the four-factor measurement structure (χ^2^/df = 2.8, CFI = 0.95, TLI = 0.94, RMSEA = 0.07), with composite reliability values ranging from 0.82 to 0.88 and average variance extracted from 0.52 to 0.61. Awareness and perceived school support together explained 36% of the variance in attitudes toward supporting student mental health (R^2^ = 0.36), with school support emerging as the dominant predictor (*β* = 0.541, *p* < 0.001). Structural equation modelling further demonstrated that self-efficacy for supporting mental health partially mediated the effects of both awareness (standardized indirect effect *β* = 0.015) and perceived school support (standardized indirect effect *β* = 0.096) on attitudes, with the strongest bivariate correlation observed between self-efficacy and perceived school support (*r* = 0.625, *p* < 0.01). Demographic analyses indicated that female teachers reported significantly higher awareness, self-efficacy, and perceived school support than male teachers (all *p* < 0.05); teachers holding a PhD scored significantly higher than master’s and bachelor’s degree holders on awareness, self-efficacy, and school support; and teachers with 11–15 years of experience consistently outperformed less experienced peers across all four variables.

In the UAE, Opoku et al. (2024) found that 244 teachers were neutral or ambivalent regarding their competence in implementing trauma-informed practices (overall M = 3.60 on a 5-point scale, where 4.0 indicated high competence). The lowest-rated domain was trustworthiness and transparency (M = 3.30), while the highest was safety (M = 3.76). Only 14% of participants had received training in trauma management. Training status was the strongest single predictor of trauma-informed competence (β = −0.56, *p* = 0.001) in a hierarchical regression model; the full model explained 51% of the variance. Younger teachers (aged 20–40) scored significantly higher than older teachers (aged 41+) (*p* = 0.001, η^2^ = 0.08), and public school teachers scored higher than private school teachers (*p* = 0.004).

Across the four practitioner-knowledge studies, the psychometric properties of the instruments used varied. Alshehri (2026) used a psychometrically validated four-construct questionnaire with Cronbach’s α ranging from 0.79–0.83, composite reliability of 0.82–0.88, and average variance extracted of 0.52–0.61. Opoku et al. (2024) developed and validated a theory-driven scale (TTMS, α = 0.89). Alshehri (2021) developed a new instrument with acceptable subscale reliabilities (α = 0.71–0.80); and Al-Hadad (2017) used instruments without established psychometric properties, relying instead on expert-developed content validity and methodological triangulation across three data sources. None of the studies assessed follow-up knowledge retention, reported implementation fidelity, or used blinded or independent outcome assessment.

## 4 Discussion

This review identified three main findings. Within the English-language literature identified by the search, no school-delivered mental health intervention for students with ID was found in the MENA region, and the only evaluated intervention was delivered in residential welfare centres. Only 108 students with ID took part directly in the included studies. None of the four practitioner samples, comprising 1,037 respondents, reported high preparedness to deliver evidence-based mental health support. The discussion follows the review’s three research questions: the nature and extent of the evidence base, the documented mental health needs and intervention effects, and the state of practitioner preparedness.

Because four of the six studies met the eligibility criteria only in part, the strength of each conclusion depends on which studies support it. Two conclusions rest on studies with full fit: bullying victimization was strongly associated with depressive symptoms among students with mild ID in inclusive Saudi schools (Alharthi et al., 2026), and Saudi special education teachers of students with ID reported moderate awareness, self-efficacy, and perceived school support, with school support the strongest predictor of their attitudes (Alshehri, 2026). Conclusions about intervention effects rest on a single study delivered outside schools (Bana et al., 2017), and conclusions about practitioner preparedness across countries draw largely on studies of mixed disability populations (Al-Hadad, 2017; Alshehri, 2021; Opoku et al., 2024). These conclusions should be read with that limitation in mind.

The disparity between this review’s findings and those of comparable international reviews highlights the structural problem. Windsor et al.’s (2023) systematic review of psychosocial-behavioral interventions for school-aged children with ID identified 27 randomized controlled trials, drawn almost entirely from North America, Europe, and Australia. Alpuche De Lille et al. (2025) examined the same intersection across all low- and middle-income countries and found only five studies from the entire Eastern Mediterranean region, none specifically designed for students with ID in MENA school settings. The present review identified six studies, only one of which evaluated an intervention, and so confirms and extends that gap at a population-specific level.

The disparity further supports the pattern of systemic underrepresentation described in the Introduction: students with ID in MENA are largely absent from the global evidence base and simultaneously underrepresented within regional school mental health research (Hamoda et al., 2022; Khoo et al., 2024).

Methodologically, the available literature reflects an early stage of evidence development. The single intervention study was quasi-experimental; the practitioner studies were cross-sectional. No study used DSM-5 or ICD-11 diagnostic confirmation, blinded outcome assessment, or follow-up measurement. Only two of six included a social desirability or comparable safeguard.

One possible next step would be a teacher- or counsellor-led feasibility pilot with diagnostic confirmation and follow-up assessment, for example at 6–12 months. Such a pilot could also incorporate culturally adapted intervention design, family-engagement components suited to the collectivist family structures common in many MENA settings, and standardized diagnostic protocols (Akhtar et al., 2021).

The single intervention study reported short-term improvements in self-esteem following cognitive-behavioral play therapy (Bana et al., 2017), in children with mild ID in residential welfare centres. However, the absence of follow-up data, below-threshold instrument reliability, and researcher-delivered design limit the conclusions that can be drawn about durability or scalability. The correlational findings from Alharthi et al. (2026) further indicate that bullying victimization and depressive symptoms may represent priority intervention targets in inclusive settings. The observed associations, together with documented gender differences, are consistent with international evidence linking peer victimization to poor mental health outcomes in children with ID (Buckley et al., 2020; Totsika et al., 2022; Erostarbe Pérez and Iniesta Martínez, 2026). Despite these identified needs, no evaluated interventions targeted depression, anxiety, behavioral support, or social–emotional functioning.

Across four samples from three countries, no group of practitioners rated itself as confidently prepared on any evidence-based mental health support skill, although three of these samples were not specific to ID. The lowest-rated competencies clustered around the technical core of behavioral and mental health practice: hypothesis-driven functional behavior assessment in Qatar (Al-Hadad, 2017), social skills programming in Saudi Arabia (Alshehri, 2021), mental-health-specific awareness and self-efficacy in Saudi Arabia (Alshehri, 2026), and trauma-informed care in the UAE (Opoku et al., 2024).

In the UAE, prior training was associated with higher self-reported trauma-informed competence and was the strongest single predictor in the regression model (Opoku et al., 2024), yet 86% of teachers had received no such training. Because the study was cross-sectional, it cannot show that training caused higher competence. One interpretation, which would need to be tested in longitudinal or experimental designs, is that limited access to training contributes to the gap.

Alshehri (2026) found that perceived school support was the strongest predictor of teacher attitudes toward supporting student mental health, with self-efficacy partially mediating the effects of both awareness and school support, a pattern consistent with Social Cognitive Theory (Bandura, 1986). These findings suggest that professional development that builds skills without addressing school-level conditions may produce limited or short-lived change. Institutional climate, administrative backing, and accessible mental health resources within schools may therefore matter to teacher preparedness as much as individual training (Jessiman et al., 2022; Emam and F. Hendawy Al-Mahdy, 2022; Sun et al., 2023).

These workforce gaps are compounded by the broader mental health infrastructure deficit across the region. In many MENA countries, child and adolescent mental health services remain concentrated in urban tertiary settings, inaccessible to most families (Okasha et al., 2025). In this context, the school may represent the most scalable platform for supporting students with ID. The present findings suggest that school-based implementation capacity remains underdeveloped.

Two further patterns were observed. First, female teachers scored significantly higher than male teachers on social skills knowledge (Alshehri, 2021), on awareness, self-efficacy, and perceived school support (Alshehri, 2026), and on trauma-informed competence (Opoku et al., 2024). The direction of this difference was consistent across three studies from two countries, but the studies measured different constructs with different instruments, and their gender samples were markedly unbalanced. In the gender-segregated school systems of the Gulf, teacher gender may also be confounded with school and student characteristics. The pattern therefore warrants further investigation rather than firm conclusions. Second, Opoku et al. (2024) reported that classroom teachers scored higher than allied professionals, including psychologists and social workers, on trauma-informed competence, which raises questions about specialist and generalist workforce preparation in MENA school settings.

None of the included studies incorporated a family component. Because the search strategy excluded records containing parent and caregiver terms, this finding may partly reflect the search method, and school-based studies with family involvement may have been missed. International evidence supports parent-mediated components and home–school collaboration as elements of effective intervention (Windsor et al., 2023; Leo et al., 2025), and the role of families in school-based mental health support for students with ID in MENA remains a question for future reviews.

### 4.1 Limitations of the study

Several limitations should be considered. First, the review was restricted to English-language publications, and three potentially relevant reports were excluded because their full texts were available only in Persian. Relevant work published in Arabic, Persian, Turkish, or French may therefore have been missed, and the findings describe the English-language evidence identified by this search rather than all research conducted in the region. Second, the search strategy had two weaknesses: it excluded records containing the terms mothers, fathers, and caregivers, which may have removed school-based studies with a family component, and its geographic block omitted Mauritania, Somalia, Djibouti, and Comoros and the variant terms Emirates, Saudi, and Türkiye. Third, the exclusion of non-indexed grey literature may have limited the identification of practice-based evidence. Fourth, the review was conducted by a single author, and no second reviewer independently verified screening or data charting, although the protocol specified independent verification of a 20% sample. Screening and charting were also assisted by large language model tools; the author made all final decisions, but errors in tool-assisted screening cannot be ruled out. Fifth, the Concept criterion was expanded during full-text screening, a deviation from the registered protocol reported in Section 2.2, and four of the six included studies met the eligibility criteria only in part. Three practitioner studies drew on mixed disability populations, which limits the specificity of practitioner-preparedness findings for students with ID. Sixth, one included study (Alshehri, 2026) was authored by the author of this review. It was screened and charted using the same criteria as all other studies, but its inclusion should be considered when interpreting the findings. Finally, the included studies were concentrated in Gulf countries and Iran, which limits generalizability to other MENA contexts, particularly lower-resourced or conflict-affected settings.

### 4.2 Conclusion

This scoping review is one of the first to map the intersection of intellectual disability, mental health support, and the MENA region, with particular attention to school and practitioner contexts. The English-language evidence it identified is small, heterogeneous, and geographically limited, with no school-delivered intervention, limited direct participation of students with intellectual disability (ID), and recurring gaps in practitioner preparedness.

From 210 records screened in the initial search and one further study identified by the update search, six studies were included, two of which met all eligibility criteria. Across these studies, 108 students with ID took part directly. The evidence base therefore remains underdeveloped relative to the known mental health needs of this population.

The findings suggest three priorities for future research, training, and policy. These are proposals informed by the review rather than conclusions it establishes. First, culturally adapted mental health programmes for students with ID could be piloted in the region, including in Gulf states, where educational infrastructure and workforce qualifications are relatively strong. Such programmes should be feasible for delivery by trained teachers, school counsellors, or allied professionals and could include diagnostic confirmation, fidelity monitoring, and follow-up assessment. Second, teacher preparation and professional development programmes could integrate mental health literacy, self-efficacy development, trauma-informed practice, and guidance on institutional support pathways into special education curricula. Third, regional school mental health frameworks, including those led by the WHO Eastern Mediterranean Regional Office, could explicitly include students with disabilities as a priority population (Hamoda et al., 2022).

## Data Availability

The original contributions presented in the study are included in the article/supplementary material, further inquiries can be directed to the corresponding author.
